# Microbiota of Cow’s Milk with Udder Pathologies

**DOI:** 10.3390/microorganisms9091974

**Published:** 2021-09-17

**Authors:** Mariya V. Gryaznova, Mikhail Y. Syromyatnikov, Yulia D. Dvoretskaya, Sergey A. Solodskikh, Nikolay T. Klimov, Vitaliy I. Mikhalev, Vitaliy I. Zimnikov, Evgeniy V. Mikhaylov, Vasily N. Popov

**Affiliations:** 1 Laboratory of Metagenomics and Food Biotechnology, Voronezh State University of Engineering Technologies, 394036 Voronezh, Russia; mariya-vg@mail.ru (M.V.G.); dyd16@mail.ru (Y.D.D.); s.solodskih@gmail.com (S.A.S.); pvn@vsuet.ru (V.N.P.); 2 Department of Genetics, Cytology and Bioengineering, Voronezh State University, 394018 Voronezh, Russia; 3 FSBSI All-Russian Veterinary Research Institute of Pathology, Pharmacology and Therapy, 394061 Voronezh, Russia; vetklimov@gmail.com (N.T.K.); mikhalevvit@yandex.ru (V.I.M.); ivanovich.vitalick@yandex.ru (V.I.Z.); voronezh81@rambler.ru (E.V.M.)

**Keywords:** microbiome, udder pathologies, sequencing, mastitis, 16S rRNA

## Abstract

Mastitis is the most common disease for cattle, causing great economic losses for the global dairy industry. Recent studies indicate the multi-agent and microbiome diversity of this disease. To understand the nature of mastitis and investigate the role of the microbiome in the development of pathologies in the udder of bovines, we performed NGS sequencing of the 16S rRNA gene of cow’s milk with pathologies of the udder. The obtained data show a significant increase in the *Cutibacterium*, *Blautia*, *Clostridium sensu stricto 2*, *Staphylococcus*, *Streptococcus* and *Microbacterium* genera for groups of cows with udder pathologies. Increasing relative abundance of the *Staphylococcus* and *Streptococcus* genera was associated with subclinical mastitis. Our data show that a relative increase in abundance of the *Staphylococcus* and *Microbacterium* genera may be an early sign of infection. We have shown, for the first time, an increase in the *Colidextribacter*, *Paeniclostridium* and *Turicibacter* genera in groups of cows with mastitis. These results expand our understanding of the role of the microbiome in the development of bovine mastitis.

## 1. Introduction

Mastitis is the most common inflammatory disease of the mammary gland of cattle, detected by an increase in the number of somatic cells (SCC) or visible abnormalities in milk [1]. The clinical manifestation is characterized by physical changes in the gland, which lead to a deterioration in the quality of milk and a decrease in its volume [2]. In contrast, in subclinical mastitis, visual symptoms and signs are absent, but there is an increase in milk SCC, which reduces the value of milk [3,4].

Mastitis is a major production and economic burden in the global dairy industry [5,6]. The main consequences of mastitis include reduced milk yield, milk illiquidity due to antibiotic residues, veterinary costs, culling of chronically infected cows, and accidental death of cows [7]. Moreover, mastitis has serious zoonotic potential associated with the release of bacteria and their toxins into milk [8]. Although many bacteria can cause mastitis, until recently the disease was mainly associated with *Streptococcus* spp. or *Staphylococcus* spp. However, recent research suggests that mastitis can be a multi-agent disease, in contrast to the generally accepted concept that mastitis is usually caused by a single pathogen [9,10]. This is indicated by the fact that the milk of cattle with clinical mastitis (CM) is a source of complex microbial communities with a wide diversity [11,12]. The most frequently isolated pathogens are *Staphylococcus aureus*, *Escherichia coli*, *Klebsiella* spp., *Streptococcus* spp., *Mycoplasma* spp., *Enterobacter* spp., *Bacillus* spp. and *Corynebacterium* [13,14,15].

Rapid progress in high-throughput NGS technology and bioinformatics tools over the past decade has prompted a transition from clinical microbiology to the genomic characterization of the microbiome associated with infectious diseases, including mastitis [16,17]. The method of selective sequencing of the 16S rRNA gene is the most commonly used genomic research tool in studying the microbiome of bovine mastitis, and it allows the separation of more than 90% of isolates at the genus level [12].

The purpose of this study is to investigate the microbiome in milk samples, obtained from bovines with subclinical and clinical mastitis, as well as bovines with udder irritation, by sequencing the 16S rRNA gene (hypervariable region V3) on the Ion Torrent PGM platform. We also performed a comparative assessment of the microbial community among milk samples from different groups. This study can help to better understand the nature of mastitis and the role of the microbiome in this pathology, as well as contribute to the development of more effective prevention and treatment of bovine mastitis.

## 2. Materials and Methods

### 2.1. Samples

The studies were carried out on Holstein cows with milk yield for the last lactation of 6850–7630 kg. The animals were kept in a 4-row barn with 180 cows each on one farm. The animals received a totally mixed diet, calculated depending on the average productivity of the group. The animals were fed 3 times a day. Whole milk samples were taken from 26 cows with different udder pathologies and were formed into 4 study groups (Table 1). The group of cows with udder irritation included animals that did not have subclinical mastitis in two tests after 48 h using a kenotest, and the content of somatic cells in the second examination was less than 200 thousand/mL.

Milk samples were taken from cows by the “Guidelines for bacteriological examination of milk and the secretion of cow udders” (methodological guidelines 115-69, in Russia). The results of clinical observations of cows are described in Table 2.

Milk was taken from the udder quarters in compliance with the rules of asepsis; thus, the teats of the udder and the hands of the personnel were treated with a 70% alcohol solution before taking a milk sample. Samples of 5–10 mL of alveolar milk were taken at the end of milking in sterile plastic tubes. During the sampling process, under no circumstances did the udder nipple touch the edge of the tube, which was tightly closed with sterile caps afterwards. The milk samples were immediately placed in liquid nitrogen. The samples were stored at a temperature of −80 °C.

After sampling, all cows with acute catarrhal mastitis were treated with antibiotics with a wide spectrum of action-injected intracisternal. Cows with subclinical mastitis were treated by antibiotics if *Staphylococcus aureus* and/or *Staphylococcus agalactiae* were isolated from the affected quarters of the udder. All animals after the last milking were injected with orbenin DC (Zoetis, Parsippany-Troy Hills, NJ, USA) in all lobes, regardless of whether the animals had mastitis or not.

### 2.2. Isolation of DNA

DNA was isolated from milk samples using a commercial ZymoBIOMICS DNA Microprep Kit (Zymo research, Irvine, CA, USA) according to the manufacturer’s protocol.

During the DNA extraction step, an extra sample containing Milli-Q water was added as a negative control. This sample was subjected to all the same sample preparation steps as the test samples from DNA extraction to library preparation and sequencing. Negative control was added to exclude the contamination of the study samples in the laboratory. At the stage of bioinformatics analysis, we used the decontam R package to identify and visualize contaminating DNA features, allowing them to be removed and a more accurate picture of observed communities to be constructed from marker-gene and metagenomics data.

### 2.3. Amplification of the 16S rRNA Gene

We selected the variable region V3 of the 16S rRNA gene to study the microbiome using sequencing on the Ion Torrent PGM. Bacterial DNA was amplified with the universal forward 337F and reverse 518R primers [18,19].

The primer sequences were as follows:

337F: 5′-GACTCCTACGGGAGGCWGCAG-3′;

518R: 5′-GTATTACCGCGGCTGCTGG-3′.

PCR was performed using a 5X ScreenMix-HS Master Mix (Evrogen, Moscow, Russia) in the following mode: 94 °C for 4 min followed by 37 cycles of 94 °C for 30 s, 53 °C for 30 s, and 72 °C for 30 s with the final elongation at 72 °C for 5 min.

### 2.4. High-Throughput Sequencing

PCR products were purified with AMPure XP magnetic beads (Beckman Coulter, Miami, FL, USA). Sequencing libraries were prepared using the NEBNext Fast DNA Library Prep kit (New England Biolabs, Ipswich, MA, USA) by following the manufacturer’s protocol.

The quality of the sequencing libraries was evaluated using qPCR and the Library Quantification Kit Ion Torrent Platforms (Kapa Biosystems, Wilmington, MA, USA). At this stage of the study, the library corresponding to sample number 2 was excluded due to its low concentration.

After that, the libraries were mixed in equimolar volumes for emulsion PCR using the OneTouch 2 system (Thermo Fisher Scientific, Waltham, MA, USA). Sequencing was performed on the IonTorrent PGM system using the Ion PGM Hi-Q View Sequencing Kit, Ion OneTouch 2 System, and Ion PGM Hi-Q View OT2 Kit (Thermo Fisher Scientific, Waltham, MA, USA).

### 2.5. Bioinformatic and Statistical Analysis

Sequencing results were obtained in BAM format and converted to FASTQ format using SAMtools v.1.2 software [20]. Demultiplexing was done with the fastq-multx application of the ea-utils v.1.3 program package. The reads were filtered according to the reading quality based on the expected number of errors using the maximum expected error cutoff of 1.0 [21]. The samples were pooled, and unique sequences were identified before searching for the operational taxonomic units (OTUs). We searched for the OTUs using the UNOISE2 algorithm, which reduces the noise through error correction [22]. We combined all reads of the samples for generating OTUs and making an OTU table. The most important reason for pooling is the fact that it enhances the signal of abundance for correct sequences. When the samples are pooled, a sequence that appears as a singleton in one sample may also appear in another sample. Thus, it is retained and included in the OTU table. If singletons are discarded after pooling (as usually recommended to reduce spurious OTUs), then more low-abundance species will be retained compared with discarding singletons for each sample separately.

Filtration of reads, identification of unique sequences, and clusterization of searching for the OTUs were performed using the VSEARCH v.2.8.2 software. Microbial species in the samples were identified using the SILVA database v.123 (https://www.arb-silva.de, (accessed on 30 July 2021)).

To compare relative abundances between different experimental groups, we used the generalized linear modelling (GLM) method implemented in the DeSEQ2 R package [23]. In brief, the final estimation of logarithmic fold changes for each OTU performed by DeSEQ2, which is based on the gene-wise dispersion of estimates comparison. The starting point of a DESeq2 analysis is a count matrix K with one row for each taxa *i* and one column for each sample *j*. The matrix entries K*ij* indicate the size of the OTU.

In our case, a comparison between four groups was performed. It produces the design matrix where elements indicate whether a sample *j* belongs to the experimental group (clinically healthy cows or the group with subclinical mastitis) or not. *p* values for each OTU were obtained using the Wald test [24].

## 3. Results

We estimated the total abundance of all identified taxa based on the number of reads. Raw sequencing data are available in the NCBI BioProject database (BioProject ID: PRJNA736244). The most common phyla are *Actinobacteriota, Firmicutes, Proteobacteria* and *Bacteroidota.* The values of the mean abundance of phyla for each group are presented in Table 3.

**Figure 1:**
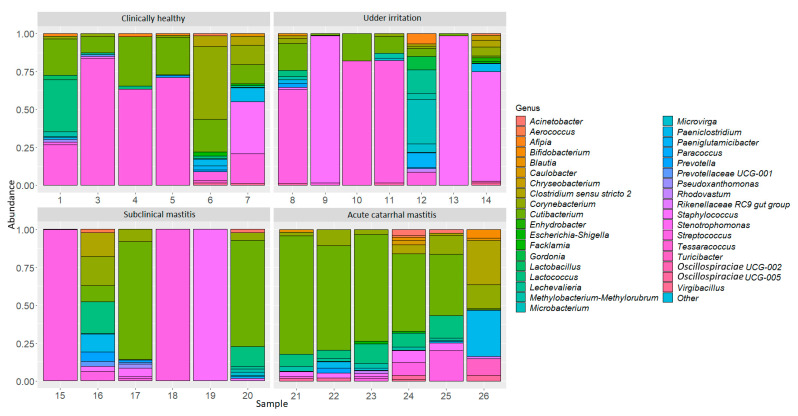
Taxonomic profile of the 37 most common bacterial genera in cow milk samples.

Relative abundance of the 37 most common genera were also evaluated in the studied groups (Figure 1). In the phylum *Actinobacteriota*, the most common genera were *Cutibacterium*, *Corynebacterium*, *Microbacterium*, *Lechevalieria*, *Paeniglutamicibacter*, *Gordonia*. In the phylum, *Firmicutes*, the most common genera were *Streptococcus*, *Staphylococcus*, *Lactococcus*, *Clostridium sensu stricto 2*, *Paeniclostridium*, *Blautia*, *Turicibacter*. In the phylum *Proteobacteria* and *Bacteroidota*, the most common genera were *Methylobacterium, Afipia, Acinetobacter, Microvirga* and *Prevotella*, and *Prevotellaceae* (Figure 1). Data showing the abundance of microorganisms for each sample are presented in the Supplementary Materials (Table S1).

**Figure 2:**
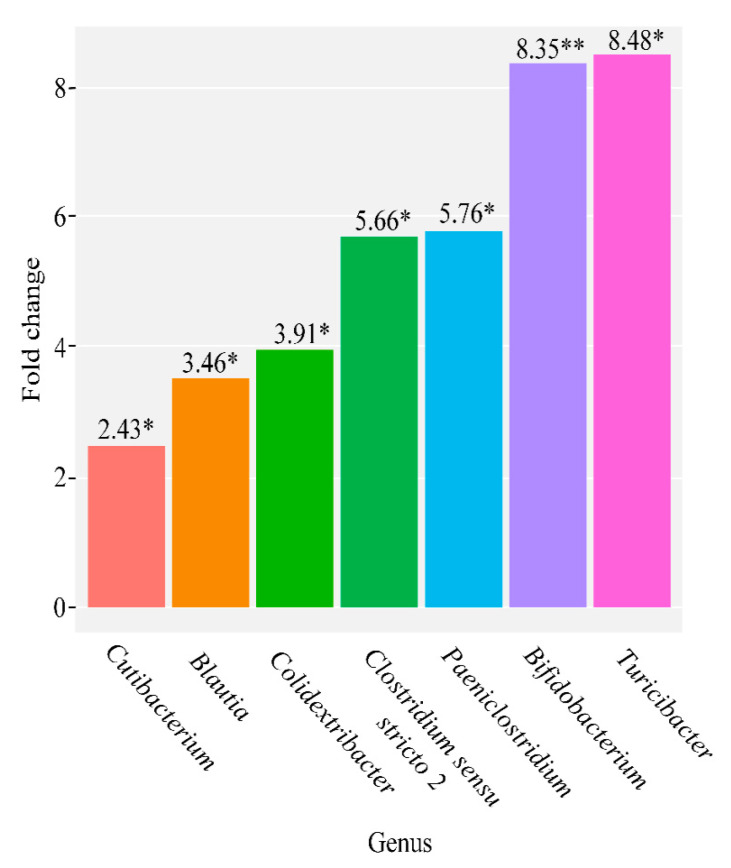
Quantitative changes in the general composition of the microbiome of milk samples taken from cows with acute catarrhal mastitis, compared to the control group (∗ *p* ≤ 0.01, ∗∗ *p* ≤ 0.001).

We have observed that, in the group of cows with acute catarrhal mastitis, the number of genera increased for *Cutibacterium* of 2.43 times (*p* = 0.0065), *Blautia* of 3.46 times (*p* = 0.0076), *Colidextribacter* of 3.91 times (*p* = 0.0047), *Clostridium sensu structo 2* of 5.66 times (*p* = 0.0038), *Paeniclostridium* of 5.76 times (*p* = 0.0056), *Bifidobacterium* of 8.35 times (*p* = 0.0005), and *Turicibacter* of 8.48 times (*p* = 0.0079), compared to the healthy control group (Figure 2).

**Figure 3:**
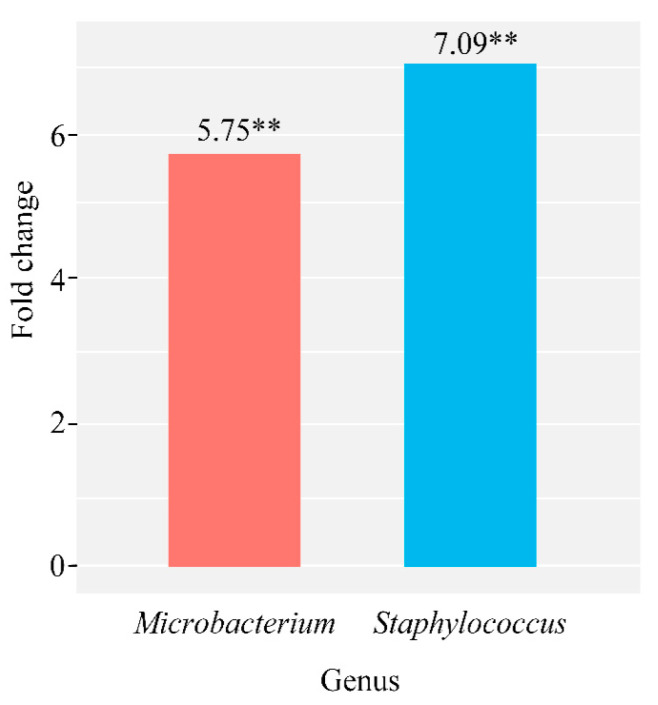
Quantitative changes in the general microbiome composition of milk samples taken from cows with udder irritation compared to the control group (∗∗ *p* ≤ 0.001).

In the group of cows with subclinical mastitis, there is a significant increase in only one genus *Staphylococcus* of 7.42 times (*p* = 0.0005), compared to the control group. We have observed an increase in the genera *Microbacterium* of 5.75 times (*p* = 0.0004) and *Staphylococcus* of 7.09 times (*p* = 0.0005) in the group of cows with pathology, when comparing the microbiome composition between the groups of cows with udder irritation and the control group (Figure 3).

**Figure 4:**
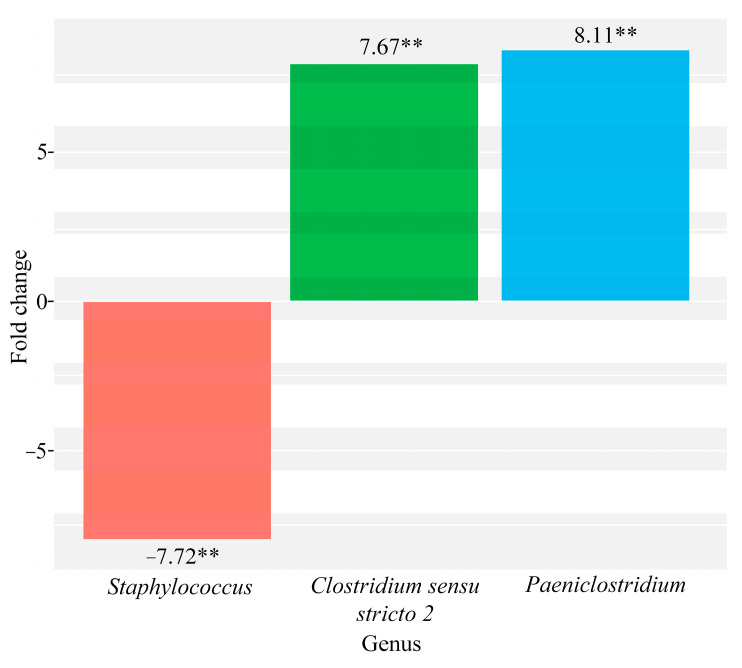
Quantitative changes in the general microbiome composition of milk samples taken from cows with acute catarrhal mastitis compared to the group with udder irritation (∗∗ *p* ≤ 0.001).

We also obtained evidence that there are significant differences in the composition of the microbiota between groups with different udder pathologies. Thus, a significant decrease is revealed for the genus *Staphylococcus* of 7.72 times (*p* = 0.0002), while an increase is shown for the genera *Clostridium sensu structo 2* of 7.67 times (*p* = 4.99 × 10^−5^) and *Paeniclostridium* of 8.11 times (*p* = 0.0001) in the group of cows with acute catarrhal mastitis compared to the group with udder irritation (Figure 4).

**Figure 5:**
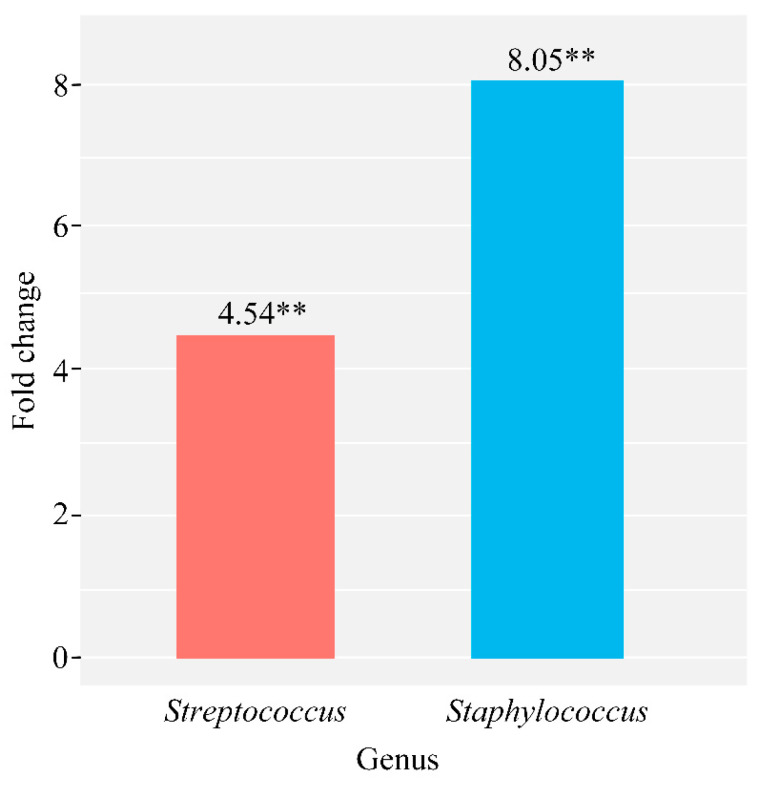
Quantitative changes in the total microbiome composition of milk samples taken from cows with subclinical mastitis compared to acute catarrhal mastitis (∗∗ *p* ≤ 0.001).

Additionally, microbiome differences have been observed between groups with subclinical mastitis and acute catarrhal mastitis, characterized by an increase in *Staphylococcus* of 8.05 times (*p* = 0.0002) and *Streptococcus* of 4.54 times (*p* = 2.97 × 10^−6^) in the subclinical mastitis group (Figure 5).

## 4. Discussion

Our research is aimed at examining the differences in the milk microbiome of healthy cows and cows with various udder pathologies, including mastitis. Significant differences are found in the composition of the microbiome, compared to the healthy group.

In the group with acute catarrhal mastitis, we observed a significant increase in the *Cutibacterium* genus in comparison with the control group. The *Cutibacterium* genus (formerly known as *Propionibacterium*) is usually one of the dominant cultured bacteria and is present on all mucosal surfaces, including in the digestive tract, which is most likely to be an important source of these bacteria for the breast environment [25]. It is also known that these obligate anaerobes have always been extracted, together with microorganisms, classically causing mastitis, which is also demonstrated in our study (Figure 1) [26]. Interestingly, in a study by Oikonomou et al., the bacteria of the *Trueperella* genus were highly significant in the development of mastitis [9]. However, in our study, bacteria of this genus were found in only one milk sample from a cow with acute catarrhal mastitis in an insignificant amount (less than 1%) (Table S1).

Bacteria of the genus *Blautia* are associated with anti-inflammatory conditions and the production of regulatory T cells [27]. We suppose that the increase in the number of these bacteria in the group with acute catarrhal mastitis may be associated with the course of the inflammatory disease and the activation of T-cell immunity (Figure 2).

Our study has found a significant increase in the genus *Colidextribacter* in milk samples taken from cows with acute catarrhal mastitis. Only one member of this genus is known. It has anaerobic properties and a Gram-negative cell wall. It was isolated for the first time from the large intestine of an obese patient and identified in 2016 [28]. Due to the lack of literature data, at the moment we cannot demonstrate how exactly the genus *Colidextribacter* is involved in the development of the clinical form of mastitis.

Our data show an increase in bacteria of the genus *Clostridium sensu stricto 2* in the group of cows with acute catarrhal mastitis, both in comparison with the control group (Figure 2) and the group with udder irritation (Figure 4). The *Clostridium sensu stricto 2* genus (also known as *Hathewaya*) is known to include proteolytic and pathogenic anaerobic organisms. Previously, bacteria of this genus have been isolated from soil, the lower digestive tract of some animals, infected cattle, water buffaloes, human faeces, human clinical specimens, including blood, peritoneal fluid, pleural fluid, and lung biopsy for pulmonary infections [29]. In addition, it is known that members of this genus are responsible for causing gas gangrene. They are also found as a part of mixed infections, moreover they are associated with the development of metritis [30]. Thus, the data obtained may indicate that this bacterial genus has high pathogenicity and plays a significant role in the development of acute catarrhal mastitis in cattle.

In our study we have identified, an increase in the genus *Paeniclostridium* in the group of cows with acute catarrhal mastitis compared to the group with udder irritation (Figure 4) and the healthy group (Figure 2). It is known for certain that representatives of the genus *Paeniclostridium* are widespread in nature and have a variety of physiological characteristics. Some species of this genus of bacteria are often found in wounds, more often in association with other anaerobic and aerobic microorganisms. The virulence of these bacteria for animals is due to β-toxin, which has high biological activity and specificity. These microorganisms are often isolated in diseases, the symptoms of which are characteristic of other clostridiosis, for example, sudden death in sheep, acute abomazitis in cows and lambs, hemorrhagic enteritis, gangrenous lesions of the reproductive tract of newborn cows [31]. Thus, based on previous studies, as well as on our data, we suppose that representatives of the genus *Paeniclostridium* can be related to the clinical form of mastitis.

Members of the genus *Bifidobacterium* are probiotic microorganisms that have a beneficial effect on the host organism. Taking into consideration this fact, we have assumed that this genus would be reduced in groups of cows with udder pathologies. The *Bifidobacterium* genus is one of the markers of “healthy microbiota” and is usually reduced in cows with udder pathology [12,32]. However, we have obtained the opposite data, according to which the genus *Bifidobacterium* has significantly increased in the group of cows with acute catarrhal mastitis, compared to the healthy group (Figure 2). We found a study in which the microbiota of milk and faecal samples from cows with mastitis was compared. The researchers have found out that the nature of changes in the microbial community of faeces in cows with mastitis was similar to that in milk, characterized by a general increase in the number of mastitis pathogens and a decrease in *Lactobacillus* and their members (*L. salivarius, L. sakei, L. ruminis, L. delbrueckii, L. buchneri* and *L. acidophilus*). At the same time, the genus *Bifidobacterium* did not correlate with the microbial community of milk and SCC in cows with mastitis [33]. Thus, literature data and our findings suggest that the biological significance of the genus *Bifidobacterium* in connection with bovine mastitis requires a thorough analysis.

According to our data, the *Turicibacter* genus increased 8.48 times in samples of cows with acute catarrhal mastitis compared to the healthy group. Recent studies based on the analysis of the 16S rRNA gene and ribosomal intergenic spacers indicate the presence of *Turicibacter* bacteria in the rumen and faeces of cattle [34]. It is also reported that *Turicibacter* is present in the intestines of pigs, rats and insects, as well as dairy wastewater and whole milk [35]. Since only one species has been isolated (*Turicibacter sanguinis*), the physiological diversity of this genus is unknown [36]. However, since the isolated strain is a suspected pathogen, there is a possibility that *Turicibacter* bacteria, which is present in farm animals, can cause infections or some other deleterious effect on the gastrointestinal tract, which is consistent with our study.

The *Staphylococcus* and *Streptococcus* genera are major contributors to the development of mastitis in cattle [37,38]. One of the most important etiological agents of bovine mastitis is *Staphylococcus* *aureus*, which is mainly associated with subclinical infection that is persistent and can easily recur [39,40]. Our data also demonstrate an increase in bacteria of the genus *Staphylococcus* in the group with subclinical mastitis, both in comparison with the healthy group and the group with acute catarrhal mastitis (Figure 4). Our data confirm the leading role of this genus in the development of the subclinical form of the disease. In addition, we have also found an increase in *Staphylococcus* in the group of cows with udder irritation (Figure 3). The data obtained may indicate that the colonization of *Staphylococcus* occurs in the outer region of the teat duct and firstly leads to irritation of the udder, which later turns into an inflammatory process, the result of which is serious damage to the epithelial cell of the mammary gland and a subclinical form of mastitis developing [41].

In addition to the increase in bacteria of the genus *Staphylococcus*, which are the trigger mechanism for the development of udder irritation, which subsequently turns into a subclinical form of mastitis, the significant increase in bacteria of the *Microbacterium* genus is also observed in the group of cows with udder irritation (Figure 3). Bacteria of the genus *Microbacterium* are members of the *Corynebacterium*, which are often isolated from milk taken from infected mammary glands of dairy cows, in addition, their increased number is associated with a decrease in milk yield [42]. However, little is known so far about the epidemiology of the genus *Microbacterium*. We consider that a significant increase in the genus *Microbacterium* in the group of cows with udder irritation may be caused by infection of the cow’s mammary gland.

## 5. Conclusions

Our data show that the milk microbiome of cows with udder pathologies differs significantly from the milk microbiome of healthy cows. The role of bacteria, such as *Cutibacterium, Blautia, Clostridium sensu stricto 2*, *Staphylococcus, Streptococcus* and *Microbacterium* in groups with various udder pathologies and their association with the development of inflammation and mastitis has been identified. In addition, our study has revealed that the *Staphylococcus* and *Streptococcus* genera are associated with subclinical mastitis. We also suggest that an increase in the genera of *Staphylococcus* and *Microbacterium* may be early indicators of infection, which firstly leads to irritation of the udder, and then to an inflammatory process that turns into subclinical mastitis.

Moreover, we would like to note that for the genus *Colidextribacter*, *Paeniclostridium* and *Turicibacter* our study has shown the association with cattle udder diseases for the first time. Additionally, we attained results for the *Bifidobacterium* genus. Our data show an increase in the *Bifidobacterium* genus in the group of cows with acute catarrhal mastitis.

These results expand our understanding of the role of the microbiome in the development of pathologies of the udder of cattle and in the future may help to create methods for mastitis prevention and treatment. Molecular methods may be developed for the rapid and early identification of the bacteria, which are associated with udder irritation and mastitis, according to our research.

## Figures and Tables

**Table 1:** Milk samples.

Sample Number	Clinical Diagnosis
1–7	Clinically healthy
8–14	Cows with udder irritation
15–20	Cows with subclinical mastitis
21–26	Cows with mastitis catarrhalis acuta (acute catarrhal mastitis)

**Table 2:** Clinical observations of cows.

Indicators	Group of Animals			
	Clinically Healthy	Cows with Udder Irritation	Cows with Subclinical Mastitis	Cows with Acute Catarrhal Mastitis
Temperature, °C	37.9 ± 0.8	37.8 ± 0.9	38.4 ± 1.2	39.8 ± 1.3
Pulse, beats/min	75.8 ± 5.1	77.3 ± 4.9	80.4 ± 5.8	82.3 ± 6.7
Breath, breathing movement/min	14.5 ± 1.1	14.9 ± 1.3	15.9 ± 1.1	16.6 ± 1.3
Reaction with Keno™ test *	**−/−**	+/−	**+/+**	not carried out
Sediment test	10-2.1 0.0	10-2.0 0.0	10-1.5 0.0	10-0.5 1.0
Somatic cells, thousand/mL	<200	>400/<200	>1000	>3500
Milk properties	No changes	No changes	No visible changes	Watery with casein clots

Note: first study/second study. * Keno™ test (CID LINES, Ieper, Belgium).

**Table 3:** Relative abundance of bacterial phyla in the studied groups.

Phyla	Mean Abundance	SD	Group
*Actinobacteriota*	0.2852	±0.1655	Clinically healthy
*Bacteroidota*	0.0482	±0.0273	
*Firmicutes*	0.5538	±0.1603	
*Proteobacteria*	0.1115	±0.0452	
Other	0.0002	±0.0008	
*Actinobacteriota*	0.1712	±0.2073	Udder irritation
*Bacteroidota*	0.0446	±0.0318	
*Firmicutes*	0.6790	±0.3069	
*Proteobacteria*	0.1497	±0.1079	
Other	0.0006	±0.0019	
*Actinobacteriota*	0.5419	±0.2424	Subclinical mastitis
*Bacteroidota*	0.0969	±0.0755	
*Firmicutes*	0.6202	±0.4266	
*Proteobacteria*	0.1032	±0.0427	
Other	0.0010	±0.0022	
*Actinobacteriota*	0.5144	±0.1782	Acute catarrhal mastitis
*Bacteroidota*	0.0333	±0.0125	
*Firmicutes*	0.3393	±0.2169	
*Proteobacteria*	0.1201	±0.0541	
Other	0.0014	±0.0028	

## Data Availability

Sequencing data are available in NCBI BioProject database (BioProject ID: PRJNA736244).

## Supplementary Materials

The following are available online at https://www.mdpi.com/article/10.3390/microorganisms9091974/s1, Table S1: The abundance of microorganisms for the samples.
